# The Diagnosis of Paternity

**Published:** 1914-05-02

**Authors:** 


### The Diagnosis of Paternity.

As everyone knows, it is a wise child that knows its own father; but this knowledge (if the anticipations of some Continental scientists come true) will no longer be "a secret only known to his mother." Drs. Mayoral and Jiminez have contributed to *La Médecine Scientifique* (according to a digest in the *American Journal of Clinical Medicine*) their researches into certain blood reactions which enable them, they believe, to pronounce definitely whether a woman is or is not pregnant. This feat, as a matter of fact, other workers have claimed to have accomplished already: and, on the face of it, there is nothing so very remarkable about it. But Drs. Mayoral and Jiminez do not stop there, for they actually believe that they can also diagnose whether any given man is or is not the father of the fœtus which the expectant mother is carrying. The matter is still in an experimental stage, and it would be premature to suggest that positive conclusions can be drawn; but certainly the published statements are extraordinarily interesting, and the possible bearings of this new development upon social and ethical problems need no emphasis here. Briefly, red blood corpuscles from a man, made into an emulsion with an isotonic solution of salt, when injected under the skin of a woman pregnant by that man, cause an erythematous slightly painful reaction around the site of injection within six hours. No reaction ensues if the woman has been impregnated by some other man.

